# Major Phytocannabinoids and Their Related Compounds: Should We Only Search for Drugs That Act on Cannabinoid Receptors?

**DOI:** 10.3390/pharmaceutics13111823

**Published:** 2021-11-01

**Authors:** Leontina Elena Filipiuc, Daniela Carmen Ababei, Teodora Alexa-Stratulat, Cosmin Vasilica Pricope, Veronica Bild, Raluca Stefanescu, Gabriela Dumitrita Stanciu, Bogdan-Ionel Tamba

**Affiliations:** 1 Advanced Research and Development Center for Experimental Medicine (CEMEX), Grigore T. Popa University of Medicine and Pharmacy, Universitatii Street, 16, 700115 Iasi, Romania; leontina.filipiuc@umfiasi.ro (L.E.F.); teodora.alexa-stratulat@umfiasi.ro (T.A.-S.); cosmin-vasilica-d-pricope@d.umfiasi.ro (C.V.P.); veronica.bild@gmail.com (V.B.); raluca.stefanescu@umfiasi.ro (R.S.); bogdan.tamba@umfiasi.ro (B.-I.T.); 2 Department of Pharmacology, Clinical Pharmacology and Algesiology, Grigore T. Popa University of Medicine and Pharmacy, Universitatii Street, 16, 700115 Iasi, Romania; 3 Pharmacodynamics and Clinical Pharmacy Department, Grigore T. Popa University of Medicine and Pharmacy, Universitatii Street, 16, 700115 Iasi, Romania; dana.ababei@gmail.com; 4 Medical Oncology-Radiotherapy Department, Grigore T. Popa University of Medicine and Pharmacy, University Street, 16, 700115 Iasi, Romania

**Keywords:** phytocannabinoids, cannabigerol, cannabidiol, tetrahydrocannabinol, synthetic cannabinoids, cannabinoid receptors, endocannabinoid system, pharmacology

## Abstract

The most important discoveries in pharmacology, such as certain classes of analgesics or chemotherapeutics, started from natural extracts which have been found to have effects in traditional medicine. Cannabis, traditionally used in Asia for the treatment of pain, nausea, spasms, sleep, depression, and low appetite, is still a good candidate for the development of new compounds. If initially all attention was directed to the endocannabinoid system, recent studies suggest that many of the clinically proven effects are based on an intrinsic chain of mechanisms that do not necessarily involve only cannabinoid receptors. Recent research has shown that major phytocannabinoids and their derivatives also interact with non-cannabinoid receptors such as vanilloid receptor 1, transient receptor ankyrin 1 potential, peroxisome proliferator-activated receptor-gamma or glitazone receptor, G55 protein-coupled receptor, and nuclear receptor, producing pharmacological effects in diseases such as Alzheimer’s, epilepsy, depression, neuropathic pain, cancer, and diabetes. Nonetheless, further studies are needed to elucidate the precise mechanisms of these compounds. Structure modulation of phytocannabinoids, in order to improve pharmacological effects, should not be limited to the exploration of cannabinoid receptors, and it should target other courses of action discovered through recent research.

## 1. Introduction

### 1.1. Phytocannabinoids

The use of *Cannabis* has a long history, and the plant has been known for its medicinal and recreational properties for several thousand years [1,2]. There is evidence that *Cannabis* was cultivated and used for various purposes by many ancient civilizations spread far and wide: the Chinese used it both for infectious and musculoskeletal disorders and to balance and harmonize the mind and the body; the Greeks used it in funeral rituals; Indian warriors consumed it in various forms for its psychoactive and analgesic properties [3,4].

The *Cannabis* plant is an annual dioeciously flowering plant, belongs to the Cannabaceae family, and includes the species *Sativa*, *Ruderalis*, and *Indica*. In the Indian Peninsula and Central Asia, it belongs to the indigenous flora, while in the equatorial regions, it does not grow naturally, and thus it is cultivated to be used for different purposes. The two main forms in which *Cannabis* is used rudimentarily are represented by dried flower bulbs (marijuana) and pieces of resin (hashish) [5].

The unique characteristics of each variety of *Cannabis* come from the presence of three types of molecules with biological activity: cannabinoids, flavonoids, and terpenoids, which, in different proportions, modulate the potency of the psychoactive effect. The last classification of phytocannabinoids was established in 2012, when it was found that the number of constituents identified in *Cannabis* was 545, from which more than 100 were phytocannabinoids. These compounds have been isolated from the resin produced by the female plants, of which the most studied are tetrahydrocannabinol (THC, with the two major compounds Δ8-THC and Δ9-THC), cannabidiol (CBD), and cannabigerol (CBG) [6,7]. The other natural cannabinoids derived from C. sativa are classified into seven more classes: cannabinol (CBN), cannabichromene (CBC), cannabinodiol (CBND), cannabielsoin (CBE), cannabicyclol (CBL), cannabitriol (CBT), and miscellaneous types [8].

Phytocannabinoid compounds have a common chemical characteristic, which is the terpeno-phenolic structure with 21 carbon atoms. This group is further classified into 11 different subclasses listed in Table 1.

The discovery of these compounds led to the identification of cannabinoid receptors (CB1 and CB2) and endogenous ligands of the endocannabinoid system. There is evidence that this system plays an essential role in many normal physiological processes, such as memory, cognition, learning, motor control, anxiety, appetite, sleep, lipogenesis, fertility, formation of insulin and muscle fibers, vasomotricity, intestinal and bronchial motility, and immune modulation, but also in pathological-like pain, inflammation, and cancer [24,25,26,27,28,29,30,31,32,33,34,35,36,37,38,39,40,41,42].

The type of effect, either beneficial or harmful, was considered to be given by the way a compound acts on CB receptors (stimulation or inhibition) and by the substances’ individual affinity for a certain type of receptor. However, an increasing amount of data suggest that cannabinoids can interact with several types of receptors, thus potentially explaining the plethora of effects noted in preclinical and clinical studies. As such, potential new drugs should be assessed by means of more complex tests, and new methods for identifying target receptors should be used [24].

### 1.2. The Endocannabinoid System

Despite the initial beliefs that cannabinoids have non-specific binding sites due to their lipophilic nature, research in the area of mapping binding sites has identified several G protein-coupled receptors that interact with cannabinoids, two of which have been studied extensively and are considered canonical receptors: cannabinoid receptor 1 (CB1R) and cannabinoid receptor 2 (CB2R) [24]. Additionally, several endogenous ligands of the cannabinoid receptors have also been identified, 2-arachidonoylglycerol (2-AG) and N-arachidonoylethanolamine (anandamide) (AEA) being the best-known signaling lipids of this class. As such, it is currently widely accepted that the endocannabinoid system consists of cannabinoid receptors, endogenous cannabinoids, and enzymes responsible for endocannabinoid synthesis, transport, and degradation [43].

Cannabinoid receptors are widely expressed in different tissues and organs, including, but not limited to, the liver, the pancreas, the gonads and gametes, the skeletal muscle, the adipose tissue, and the skin [44,45,46,47,48]. However, the highest concentration of CB1R can be found in the nervous system—CB1R is highly expressed on glutamatergic, cholinergic, glycinergic, and serotonergic neurons, especially on synaptic terminals. Although not as abundant, CB2R can also be found in the CNS, especially in microglia and other cells of immune origin [49]. This wide distributon of cannabinoid receptors suggests that the endocannabinoid system is extremely complex and multifunctional, interacting with several different signaling pathways (including the dopamine and opioid pathways) and modulating a plethora of endogenous processess. Another interesting trait of CB1R that contributes to the system’s complexity is that, although CB1R is primarily expressed in the cell membrane, distinct CB1R subpopulations exist within the cell, most notably in lysosomes and in mitochondria [50], thus pointing towards a potential involvement of the cannabinoid system in even more pathological conditions than previousely suggested. Additionally, a recent body of evidence has pointed towards these receptors’ ability to form homo- and heterodimers with several types of receptors such as mu-opioid, dopamine, or adenosine A2 receptors [51], thus further increasing the complexity of the system. Of note, endocannabinoids interact with the two specific receptors described above, via the G protein, but also interact with non-cannabinoid receptors such as vanilloid receptor 1 (TRPV1), previously referred to as capsaicin receptor, transient receptor potential ankyrin 1 (TRPA1), and G55 protein-coupled receptor (GPR55), and nuclear receptors peroxisome proliferator-activated receptor alpha PPARα and peroxisome proliferator-activated receptor gamma (PPARγ or PPRARG) [26,52].

There are numerous reports regarding the biological changes that occur after administering CB1R agonists, most of which have concluded that the effects are biphasic and pleomorphic [53]. This is a two-edged sword, since the abundance of CB1Rs throughout the body is probably associated with diverse side effects that could affect various tissues and systems—the systematic activation of CB1R has been asociated with cardiovascular, digestive, and neurological side effects. As such, aiming to modulate receptor affinity and to identify as many binding sites as possible for exocannabinoids could prove to have immense therapeutic potential. It is widely agreed that CB1R binding is responsible for the cannabinoids’ psychotropic effect, which is why, in recent years, an important part of research has focused on binding CB2R with the aim of modulating pain and inflammation [54].

The second component of the endocannabinoid system consists of the CB1R and CB2R endogenous ligands. The first endogenous cannabinoid to be discovered was AEA, shortly followed by 2-AG. Despite additional data that have shown there are several other endogenous peptides and arachidonic acid derivatives that bind to CB receptors [55], most research in the field is still focused on the two aforementioned agonists. AEA is a high-affinity partial agonist of CB1R that has little to no effect on CB2R. Available data indicate it has a cannabinomimetic effect and modulates several essential processes both in the central nervous system and in the periphery [56]. It is synthesized in an on-demand manner, the best-known trigger for synthesis being an increase in intracellular Ca2^+^ concentrations [57] following a postsynaptic neuronal depolarization [58,59], as suggested by in vitro and in vivo experiments performed in the nucleus accumbens core [60]. 2-AG is a moderate-affinity full agonist of both CB1R and CB2R whose baseline levels seem to be higher than those of AEA in several tissues such as brain, spleen, or liver tissue [59]. One of its main roles involves regulating neurotransmitter release in different neurocognitive processes such as emotion and pain sensation [61]. Although there is still controversy regarding the synthesis of 2-AG, it appears it is also an on-demand Ca-dependent system similar to that of AEA. Both AEA and 2-AG levels can be modulated by several endogenous and exogenous factors such as chronic stress exposure, cortisone treatment, fasting, starvation, and pain [58,62].

Although not yet completely elucidated, the sum of enzymes and peptides involved in the synthesis, transport, and degradation of endocannabinoids and their receptors also plays an important role in this system and deserves more attention in that the modulation of the endocannabinoid tone is responsible for the main positive/negative effects on health. Among the best studied elements are N-acylphosphatidylethanolamine phospholipase, phospholipase C, diacylglycerol, lipase α or β, fatty acid amide hydrolase, and monoacylglycerol lipase [26,63].

## 2. Major Phytocannabinoids: Cannabigerol-, Cannabidiol-, and Tetrahydrocannabinol-Type Compounds

### 2.1. Structure–Affinity Relationship of Cannabinoid Receptors

The present section aims to provide information on the influence of different substituents of the resorcinyl moiety on the affinity of phytocannabinoids toward human cannabinoid receptors 1 and 2.

**Figure 1:**
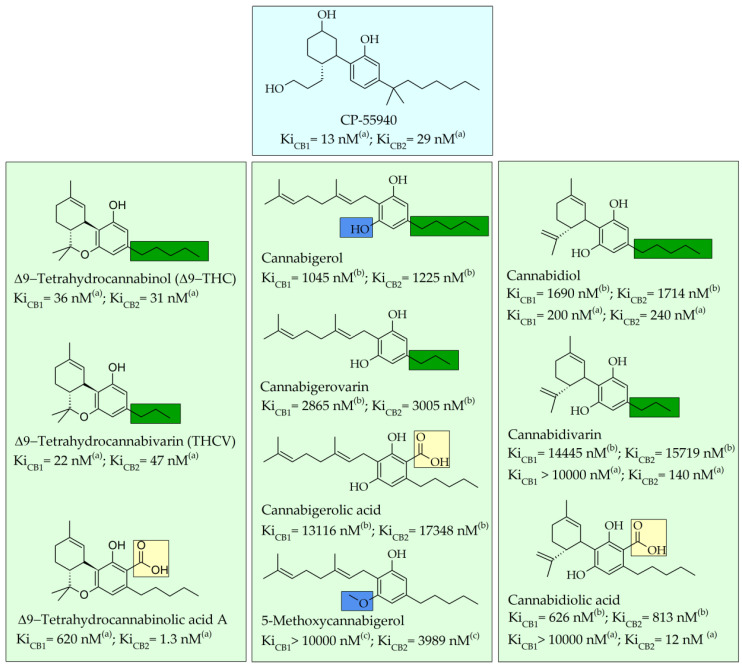
Chemical structures and binding affinities of ten major phytocannabinoids able to cause, in vitro, the displacement of synthetic [^3^H]CP55940 bound to human cannabinoid receptors 1 or 2 both embedded in cellular membranes. The binding affinities are reported according to the published data available in the following scientific articles: ^a^ [65]; ^b^ [70]; ^c^ [68].The chemical structures were prepared with ACD/ChemSketch, and the three classes of phytocannabinoids are included in separate inserts. The substituents of the resorcinyl moiety whose replacements cause a change in affinity constants (Ki) are highlighted.

Taking into consideration the high number of phytocannabinoids described in the scientific literature [64], only the binding affinities of the compounds included in the cannabigerol-type compounds, cannabidiol-type compounds, and tetrahydrocannabinol-type compounds are presented in the following paragraphs. Additionally, another limitation commonly encountered in this research field is that only few studies contain the determination of the binding affinities of multiple cannabinoids using identical assay conditions within the same laboratory [65,66,67,68,69]. These studies employ a synthetic cannabimimetic compound denoted as CP55940 prepared as radioligand [^3^H]CP55940 which possesses a high affinity for CB1 and CB2 receptors (Figure 1). Cellular membranes containing either CB1 or CB2 receptors obtained from different cellular cultures were used to assess the affinity constant of phytocannabinoids based on radioligand displacement.

For tetrahydrocannabinol-type compounds, Δ9-*trans*-tetrahydrocannabivarin containing a three-carbon alkylic substituent of the resorcinyl moiety instead of the five-carbon substituent of Δ9-*trans*-tetrahydrocannabinol showed a slightly increased affinity toward CB1 and a slightly diminished affinity toward CB2 [65]. The carboxyl group present in the natural compound Δ9-*trans*-tetrahydrocannabinolic acid A, adjacent to the hydroxyl group, causes a decrease in the affinity toward CB1 and raises the affinity toward CB2 [65].

Within the cannabigerol class of phytocannabinoids, one of the following changes leads to a decrease in the affinity of the compound in comparison with cannabigerol: (i) reduction in the alkylic side chain from five to three carbon atoms, (ii) the presence of the carboxyl group adjacent to the hydroxyl group of the resorcinyl moiety, and (iii) the presence of the methoxy moiety [67].

The analysis of the major compounds from the cannabidiol class indicates the results of different studies are difficult to correlate, probably due to the difference in the assay conditions. However, a decrease in the affinity for CB1 can be observed for canabidivarin and cannabidiolic acid in comparison with cannabidiol [65,67].

Taking into account these results, the most likely model of interaction between phytocannabinoids and cannabinoid receptors CB1 and CB2 involves noncovalent phenyl–phenyl interaction between the resorcinyl moiety and a phenylalanine residue present in the active site of the cannabinoid receptors. Delocalization of the π-electrons of the resorcinyl moiety would be the cause of the strong effect of lowering the affinity for CB1 in the case of carboxylic phytocannabinoids. This model agrees with the recent X-ray crystallography data on the CB1 structure [71].

### 2.2. Pharmacological Effects

In preclinical and clinical studies, phytocannabinoids have shown pleiotropic effects resulting in diverse clinical applications. Table 2 summarizes all the data related to different tested targets for each family of compounds (CBG, CBD, THC).

Moreover, these three major families of compounds studied preclinically or in clinical trials in different pathologies are shown in Table 3 and Table 4.

#### 2.2.1. Cannabigerol (CBG)-Type Compounds

Cannabigerol-type compounds represent one of the most structurally diverse classes of phytocannabinoids, being the second most abundant in the *Cannabis* plant, making up 16.3% of the phytocannabinoid content, with the most important compounds being represented by: cannabigerol (CBG), cannabigerolic acid (CBGA), cannabigerovarin (CBGV), cannabigerovarinic acid (CBGVA), ortho-methyl cannabigerol, and cannabigerolic acid methyl ether [8].

Cannabigerol (CBG), a minor cannabinoid present in small amounts (<1%) in the *Cannabis* plant, serves as the direct precursor to cannabidiol (CBD) and tetrahydrocannabinol (THC), and it was the first natural cannabinoid to be synthesized [64]. The compound was purified from *Cannabis* in the same year as THC (1964) by Gaoni and Mechoulam, and, soon enough, it was found that CBG does not have the same psychotropic effects as THC [105].

Cannabigerolic acid (CBGA) is one of many minor cannabinoids produced by the *Cannabis* plant; however, CBGA is at the top of the cascade reaction that produces the three major cannabinoids: THC, CBD, and CBC. In small proportions, CBGA may convert to CBG, but most of the CBGA produced in the *Cannabis* plant converts into either THC or CBD [106].

Cannabigerivarin (CBGV) and cannabigerivarinic acid (CBGVA) were isolated by Shoyama et al. between 1975 and 1977: CBGV from the benzene extract of *Cannabis*, and CBGVA from an extract of dried leaves of Thai *Cannabis* [107,108].

Cascio et al. found that CBG binds the CB1 receptor from mouse brain membranes with Ki = 381 nM, and the CB2 receptor from CHO cells expressing the human receptor with Ki = 2.6 μM [86]. In 2014, Rosenthaler et al. obtained Ki values of 897 nM for CB1 and 153 nM for CB2 in competition assays [69]. Additionally, CBG did not produce psychotropic effects in the in vivo tests, but it did affect endocannabinoid function indirectly by inhibiting anandamide uptake, contributing to increasing the levels of anandamide [76].

In a 2020 study, it was demonstrated that CBGA has a very low affinity for both CB1R and CB2R using [3H]-CP-55940 as a ligand, and thus the compound lacks pharmacological effects due to CB receptor modulation, while CBGV was the compound with greater affinity. From this class of phytocannabinoids, CBGV remains the only one with questionable behavior regarding cannabinoid receptors because, in the same study, the authors concluded that CBGV has a complex behavior, acting as a potent agonist, through Gi and MAPK pathway activation, studied in vitro, but with the observation that, in vivo, CBGV acts as an inverse agonist of cannabinoid receptors [67].

Because these compounds showed only a marginal affinity for CB receptors, they were not taken into account in studies until recently, but it seems that they have a lot of other pharmacological effects with possible applications in the treatment of neurodegenerative diseases, inflammatory disease, cancer, anxiety, and depression [72], as well as infectious diseases, metabolic disorders, and psoriasis [64,109,110].

It was found that this class of compounds along with derivative compounds presents many different actions such as neuromodulatory, neuroprotective, anti-inflammatory, analgesic, anxiolytic, antidepressant, and antibacterial activity, or that they can be used to treat cancer, psoriasis, metabolic diseases such as diabetes and their complications, anorexia, or cancer-induced cachexia by mechanisms that do not involve CB receptors [72,74,86,111,112].

Transient receptor potential ankyrin 1 (TRPA1) is a good target for the discovery of novel medicines to treat pain because it has a primary role in nociceptive transduction and neurogenic inflammation, contributes to noxious cold sensation, and plays a role in neuropathic and inflammatory pain [113]. TRPA1 is activated by CBG with an EC50 value of 3.4 ± 1.0 µM (0.6 ± 0.1), but it was found that through cyclization, the activity on this receptor is increased, and the cyclized analog of CBG could be considered for future studies as an analgesic and anti-inflammatory agent [75].

Transient Receptor Potential Melastatin-8 (TRPM8) is a non-selective cation channel activated by cold temperature and by cooling agents, and a neuronal sensor that plays a role in cold and mechanical allodynia associated with neuropathic pain secondary to trauma. It was proved that this channel is involved in pain perception, and that TRPM8 activation or deactivation can modulate analgesia [114]. De Petrocellis demonstrated in his paper that CBG is the second most efficient TRPM8 antagonist, and he found that a “CBG-free” extract from the *Cannabis* plant (with the exact quantity of CBG that was extracted from the plant) was inactive per se, but when added to pure CBG, the activity of the CBG-enriched extract was significantly increased, and it was more efficient in antagonizing TRPM8 than pure CBG. Thus, he suggested that there might be a synergistic effect between pure CBG and some of the components of its corresponding *Cannabis* extract [72].

Transient receptor potential vanilloid-3 (TRPV3) and transient receptor potential vanilloid-type 4 (TRPV4) are indirectly involved in gastrointestinal inflammation from inflammatory bowel diseases because they function as sensors of harmless and non-harmless chemical or physical stimuli [115]. It was demonstrated on in vitro models of overexpressed TRPV3 channels (against carvacrol) and overexpressed TRPV4 channels (against 4α-phorbol-12,13-didecanoate(4α-PDD)) that CBG produced a significant TRPV3 and TRPV4 desensitization as a result of their activation, which was associated with an anti-inflammatory effect [76]. In another in vivo model of murine colitis produced by intracolonic administration of dinitrobenzene sulphonic acid (DNBS), CBG had an anti-inflammatory effect associated with the downregulation of cytokines and inducible nitric oxide synthase expression (iNOS) levels [77].

In a study from 2012, the same author measured the activity of CBGA and CBGV on in vitro models of overexpressed TRPV3 channels (against carvacrol) and overexpressed TRPV4 channels (against 4α-phorbol-12,13-didecanoate(4α-PDD)). The results suggested that these two compounds desensitize TRPV3 and TRPV4 channels at lower doses than those at which they stimulate these channels. These findings have led to the conclusion that these compounds are good candidates for studies on in vivo models of conditions involving overexpression of TRPV3 and TRPV4 channels, which involve pain and inflammation in inflammatory bowel disease (IBD) [76].

TRPV1 is activated by heat, protons, and proinflammatory cytokines and is associated with pain and inflammation. The results of several studies suggest the potential of TRPV1 agonists, antagonists, and positive allosteric modulators in the treatment of pain [104,105,106]. It seems that CBGA can stimulate human TRPV1 sufficiently in order to be a potent candidate for future studies on TRPV1-modulating effects (EC50 = 1.0–2.0 mM) to treat pain and inflammation [72].

In a 2011 study, CBG and CBGA showed inhibition of more than 30% of cyclooxygenase-1 (COX-1) and cyclooxygenase-1 (COX-2) in a concentration-dependent manner, thus having enhanced anti-inflammatory effects [62]. CBG has also been found to have anti-inflammatory action by interfering with the prostaglandin E2 (PGE2) synthesis pathways at various levels: by inhibiting the enzyme cytosolic phospholipase A2 (PLA2), which catalyzes the production of arachidonic acid from membrane phospholipids, or by inhibiting monoacylglycerol lipase (MAGL), which catalyzes the production of arachidonic acid from 2-arachidoylglycerol [25].

Animal studies (mice) have shown that CBG has antinociceptive and anti-inflammatory effects on several acute pain models such as those induced by using intraperitoneally administered formalin and carrageenan. Both models are mediated by α2-adrenoceptor because they are blocked by yohimbine, an α2-adrenoceptor antagonist [25].

In a computational model of α2A, α2B, and α2C isoforms of murine and human adrenoceptors, CBG affinity for the receptor appeared higher than that of the α2-adrenergic receptor agonist clonidine. This affinity of CBG seems to be associated with sedation and analgesia and needs future research [116].

CBG exhibits the best growth inhibition against human oral epithelioid carcinoma cell lines and fibroblasts and has antiproliferative action in mouse skin melanoma cells [117,118].

It was also shown that TRPM8 expression strongly increases in prostatic cancer, and in neuroendocrine tumor cells from pancreatic cancer and colon cancer. CBG seems to be a potent TRPM8 antagonist (IC50 = 0.16 ± 0.02), which suggests it may have a therapeutic effect in these types of cancers [72].

In another study published in 2014, it was shown that CBG inhibits the growth of colorectal cancer cells primarily through a pro-apoptotic mechanism and blocks colon carcinogenesis in vivo. This effect of CBG is associated with reactive oxygen species overproduction, and the authors suggested that CBG can be a promising curative and preventive pharmacological agent for colorectal cancer [111].

Transient receptor potential cation channel subfamily V member 1 (TRPV1), also named “capsaicin receptor”, was recently reported to be aberrantly expressed in many tumor types such as breast cancer, skin tumors, or colon cancer. It was found in several studies that its activation by capsaicin was associated with antiproliferative effects. That is why the TRPV1 channel offers new treatment possibilities for cancer [119]. In a study of the effects of cannabinoids on ionotropic TRP channels conducted by De Petrocellis in 2011, it was demonstrated that CBGA stimulated human TRPV1 enough to be a potent candidate for future studies on agents that modulate TRPV1 [72].

CBGV is the most important antagonist of TRPV2 (EC50 = 1.7 μM), enough to be considered as a potential pharmacological agent to treat cancer because the overexpression of TRPV2 has been linked to several cancer types and cell lines such as urothelial cancers, prostate cancer, breast cancer, esophageal squamous cell carcinoma, and benign hepatoma, and in hepato-carcinomas and hematological malignancies, such as myeloma or acute myeloid leukemia [72]. TRPV2 channels control multiple processes involved in cancer progression by modulating survival, cell proliferation, angiogenesis, migration, and invasion in different cancer types. Clinical data suggest there might be a direct relationship between altered TRPV2 expression and a negative prognosis [81].

A recently published article concluded that CBGA is a good candidate for colorectal cancer treatment, with increased toxic activity on colorectal cancer cells and reduced activity on normal colon cell lines. In the same study, it was found that CBGA acts synergistically with THCA against neoplastic cells, but with a CBGA fraction that is greater than the THCA fraction. The cytotoxic activity of CBGA and THCA+CBGA most often led to cell cycle arrest and apoptotic cell death for cancer cells. CBGA, THCA, and CBGA+THCA are also active on adenomatous polyps, suggesting another possible therapeutic value [120].

TRPV1 is involved in the modulation of anxiety and may have implications in the treatment of depression [121]. TRPV1 may affect the locomotor activity and plays a role in thermosensation as it can be activated by noxious heat (>42 °C) [122,123]. Due to all these findings and considering De Petrocellis’s studies on the modulatory action of CBGA on TRPV1, we can assume that CBGA could be a potent candidate for future studies on TRPV1-modulating effects to treat the neurological disorders in which these channels are involved, such as anxiety or depression [72,76].

In an experiment performed on mouse brain membranes, it was proved that CBG can activate α2-adrenoreceptors and block 5-HT1A receptors, antagonizing the 5-HT1A receptor agonist R-(+)-8-hydroxy-2-(di-n-propylamino) tetralin. The authors concluded that CBG has the ability to inhibit noradrenaline uptake in the [35S] GTPγS binding assay, the brain needs further investigation, and more in vivo experiments are required to verify that the in vitro dose is enough to also be effective in vivo without adverse effects [86].

In an in vivo model of multiple sclerosis produced with TMEV (Thaler’s murine encephalomyelitis virus), CBG and CBG-quinone showed anti-inflammatory and neuroprotective effects through the inhibition of IL-1β and IL-6 cytokines, and downregulation of PGE2 synthesis. CBG and CBG-quinone inhibited the microglia inflammatory response, protected neurons from toxic insults in vitro, and restored motor function impairment [124].

Using an in vivo model of Huntington’s disease (HD), created using nitopropionic acid (3-NPA), CBG efficacy was tested using a dose of 10 mg/kg/day administered i.p. and was shown to have neuroprotective effects by downregulating the proinflammatory markers COX-2, iNOS, IL-6, and TNF-α, by preventing neuronal degradation, downregulating disease-associated genes *SgKL* and *CD44*, and normalizing specific protein-1 levels. Clinically, specific symptoms such as dystonia and tightening of the hind limbs were visibly improved, and locomotor activity was enhanced [125].

In an in vitro model of neuroinflammation on NSC-34 motor neurons, pretreatment with CBG (7.5 μM) improved viability in treated cells through the inhibition of cell apoptosis, a reduction in IL-1β, TNF-α, IFN-γ, and PPAR-γ proinflammatory protein levels, a reduction in oxidative stress, and upregulation of Nrf-2 levels [74].

CBG blocked cell death, reduced oxidative damage, and prevented neurons from accumulating toxic β-amyloid protein, in an in vitro Alzheimer’s disease model [126].

In an in vitro study on the effect of CBGA and CBG on the aldose reductase (ALR2) enzyme, it was found that both drugs showed statistically significant ALR2 inhibitory activity by being able to interact with the major active site of the enzyme. Considering that ALR2 is a key enzyme involved in diabetic complications, the results obtained in this study may have some relevance in medicine to prevent or treat possible diabetic complications [79].

In a 2019 in silico study, a computer simulation was developed in order to assess CBGA’s role in activating peroxisome proliferator-activated receptors (PPARs) that regulate metabolism. Three phytocannabinoids, namely, CBGA, CBDA, and CBG, were thus identified as being PPARα/γ dual agonists. The study established that they act as full or partial agonists on PPARα/γ isoforms. Effects on downstream gene transcription in adipocytes and hepatocytes were also noted, further certifying their roles as functional dual agonists [80]. It is already known that peroxisome proliferator-activated receptor agonists such as rosiglitazone and pioglitazone are used to treat diabetes to improve the pathogenesis of insulin resistance and hyperglycemia; therefore, CBGA can be considered a good candidate for the treatment of diabetes complications [127].

It was found that higher doses (120 to 140 mg/kg) of GBG induce a dose-dependent increase in food intake, increase the number of meals taken, decrease the latency until the first meal, and improve locomotor activity [128]. Another study also found that pure CBG (120 mg/kg) can attenuate weight loss induced by the chemotherapy agent cisplatin [25]. Due to these findings, CBG could be useful for the treatment of feeding disorders and weight loss in cancer anorexia-cachexia syndrome [25,128].

It was found that CBG has antibacterial properties against Gram-positive bacteria, mycobacteria, and fungi [109]. Recently, the antibacterial properties of CBG were tested against various methicillin-resistant *Staphylococcus aureus* (*S. aureus*) strains of current clinical relevance, and the results were promising [110].

In a 2020 study on CBG antibacterial activity against methicillin-resistant *S. aureus* (MRSA), it was found that, in vitro, CBG acts through the disruption of the cytoplasmatic membrane of MRSA. Using an in vivo model of systemic MRSA infection in mice, CBG was shown to express antibacterial action comparable to vancomycin, at a non-toxic dose of 100 mg/kg, suggesting that CBG could be a less toxic alternative for the treatment of methicillin-resistant Gram-negative bacteria [129].

In a study on the effects of CBG in skin conditions, CBG demonstrated inhibitory action on keratinocyte proliferation in a CB1/CB2 receptor-independent manner, being a good candidate for the treatment of psoriasis [130]. Additionally, CBG acted as a transcriptional repressor controlling cell proliferation and differentiation through a mechanism that involved increasing DNA methylation on the keratin-10 gene, making CBG a good candidate for the development of novel therapeutics for skin disease [131].

#### 2.2.2. Cannabidiol (CBD)-Type Compounds

Cannabidiol (CBD) is considered to be the second phytocannabinoid in abundance after Δ9-THC, biosynthesized by the plant *Cannabis sativa*, with a defensive role against parasites that may affect the plant, being its main non-psychomimetic compound [26,27,132]. Numerous preclinical and clinical studies have reported that, due to the absence of psychoactive effects, CBD could have great potential in the treatment of symptoms characteristic of neuropsychiatric disorders (anxiety, depression, substance use disorders, dependence on various drugs, epilepsy, psychosis, Parkinson’s, Alzheimer’s, multiple sclerosis, Huntington’s disease), migraine, inflammatory diseases, rheumatoid arthritis, etc. [26,27,28,29,133,134].

The CBD acidic precursor cannabidiolic acid (CBDA) is one of the most present phytocannabinoids in European hemp [32]. Of all cannabinoid acids, CBDA seems to be the one with the weakest pharmacological activity, being studied thus far for its effects in pain, inflammation, and nausea but also for its therapeutic potential in treating breast cancer and in relieving the symptoms of Dravet syndrome [92,94,135,136].

Cannabidivarin (CBDV), isolated in 1969 by Vollner et al., is a CBD homolog that has begun to attract the attention of researchers for its pharmacological profile because it has low activity at CB1 receptors, thus lacking the psychotropic effects related to CB1 receptor activity [67,137]. Regarding CB2 receptors, the affinity of CBDV for these receptors is still under scrutiny, the results of the latest studies being contradictory. While Zagzoong et al. reported a high affinity for these receptors measured using Chinese hamster ovary (CHO) cells expressing human CB2 receptors, Navarro reported a low affinity using a heterologous system expressing human versions of CB1 and CB2 receptors. In this situation, the best way to solve this contradiction is by performing more in vivo tests on the effects of CBDV on models such as chronic and acute pain, epilepsy, or anxiety as pathologies in which the activity of CB2 receptors could be involved [65,67].

CBD pharmacokinetics are complex and variable, with low oral bioavailability due to incomplete oral absorption and high hepatic clearance, but can be greatly increased (4-fold) when combined with high-fat or high-calorie meals. Due to its low bioavailability, CBD shows a high pharmacokinetic variability with consequences on clinical response. Due to its highly lipophilic nature, it has a large volume of distribution (23–43 L/kg), is highly bound to plasma proteins (>94%), and the time required to reach the maximum plasma concentration when administered as a single dose is 3–5 h. Its plasma half-life depends on the dose and route of administration, with great variability between humans and rodents, ranging from 18 to 32 h, making dosing difficult at this time. It is excreted via feces [67,87].

The numerous effects of CBD have increased the interest in its pharmacological properties. It has a low affinity for cannabinoid receptors but can act as a negative allosteric modulator of CB receptors. Although it has a low affinity for these receptors, it is nevertheless interesting that CBD may favor increases in endogenous endocannabinoid levels by indirect mechanisms. However, its actions are much more varied: for example, it intervenes in modulating the activity of some neurotransmitter transporters such as dopamine, norepinephrine, adenosine, and glutamate [84,138].

It can be an agonist of transient receptors (TRPV1), of 5-HT1A receptor, and of PPARγ receptor, or it can modulate the activity of numerous ion channels and various enzymes as well as modulating G55 protein-coupled receptor (GPR55) [84].

CBDV has been studied in vitro and has been shown to stimulate TRPA1, TRPV1, and TRPV2 channels in a dose-dependent manner [72,88], to act as an antagonist for TRPM8 [72] channels and GPR55 [134], and to act as an inverse agonist of GPR6 [90]. CBDV may also indirectly affect CBR signaling, by inhibiting the cellular uptake of AEA, or by inhibiting diacylglycerol-lipase-α (DAGLα) [72]. In vivo, CBDV also acts as a partial agonist for dopamine D2-like receptors [139].

CBD, CBDA, and CBDV have been found to have numerous beneficial effects on the relief of various symptoms, especially in central nervous system disorders. CBD was tested in various neurological disorders such as depression and mood disorders, schizophrenia, Dravet syndrome (DS), Lennox–Gastaut syndrome (LGS), and Alzheimer’s, and in other types of pathologies such as neuropathic pain and inflammation [29,87,91,135,138,140,141]. CBDV has also become a good candidate as a therapeutic agent for neurological diseases such as epilepsy and autism spectrum disorders, Rett syndrome, ischemic strokes, or inflammatory pathologies such as inflammatory bowel disease (IBD) [91,142,143,144].

The first preclinical study showing that CBD could be effective in relieving depressive symptoms was published in 2010; in a murine (mouse) model, CBD reduced the immobility time in mice undergoing a forced swimming test, the effect being similar to that produced by antidepressants such as imipramine. The authrors concluded that the effects of CBD are most likely initiated by the activation of 5-HT (1A) receptors, receptors involved in the neurobiology of depression [85].

Based on the idea that CBD does not bind directly to CB1 receptors, the antidepressant effect may be due to indirect modulation of the endocannabinoid system in the prefrontal cortex with subsequent activation of CB1 receptors, thus promoting 5-HT(1A) activation in cortical and limbic brain regions. Both in vivo and in vitro studies have shown that chronic CBD treatment promotes hippocampal neurogenesis and synaptogenesis by increasing anandamide signaling in the hippocampus, while endocannabinoid system signaling promotes neurogenesis via CB1 and CB2 receptors. These observations may represent evidence to support CBD as a potential drug to treat mood disorders [26,84].

A recent randomized, double-blind, placebo-controlled clinical trial evaluated the acute effects of tetrahydrocannabinol, cannabidiol, and their combination on the Emotional Recognition Facial Affect Test which showed that inhalation of a 16 mg single dose improved subjects’ performance on this test by 60% in emotion intensity [145]. Other clinical studies have shown that a single dose of CBD (300/600 mg/kg) reduced anxiety in healthy volunteers during public speaking, effects that may depend on changes in brain regions involved in emotional processing [26].

In recent years, research in the field has been increasingly focused on understanding the pathophysiological mechanisms involved in drug addiction, a serious public health problem, with CBD recently being considered as a potential therapeutic approach. In this regard, a preclinical study published in 2021 investigated the possible beneficial effects of CBD on relapse symptoms after amphetamine re-exposure, drug relapse being the most difficult clinical factor to control during addiction treatment. The study was conducted on 43-day-old rats, an age that was selected as it corresponds to the adolescent period, a highly vulnerable period for the development of drug abuse conditions. CBD treatment was able to prevent amphetamine relapse behavior in rats that had previously exhibited amphetamine-conditioned place preference and modulated immunoreactivity of dopaminergic targets in the prefrontal cortex and ventral striatum, areas with major involvement in drug dependence. Amphetamine impairs dopaminergic neurotransmission by altering dopamine transport, but in contrast, this study showed that CBD was able to maintain dopamine transport levels. However, this study could not state whether CBD treatment reversed the molecular changes underlying amphetamine conditioning [27].

Other preclinical and clinical studies suggest that CBD could also be useful in treating schizophrenia, since this drug seems to exert antipsychotic effects in various animal models. Thus, CBD may restore deficiencies in prepulse inhibition (PPI) of the startle reflex. PPI is described by the response decrement that occurs when an acoustic stimulus is preceded by one below the subthreshold. In schizophrenia, this behavioral modification is proposed to reflect the impaired sensorimotor gating. Furthermore, the disruption of PPI may also be induced by compounds that facilitate the inhibition of glutamatergic or dopaminergic neurotransmissions such as N-methyl-d-aspartate (NMDA) receptor antagonist, amphetamine (AMPH), or dizocilpine (MK-801) [132]. Other studies showed that CBD reduced apomorphine-induced stereotypy and amphetamine- and ketamine-induced hyperlocomotion, and enhanced extracellular dopamine levels in the nucleus accumbens, with doses required for its antipsychotic action being higher compared to those used to induce anxiolytic effects [26].

Epidiolex, a prescription medicine that contains CBD, was the first drug approved in June 2018 by the Food and Drug Administration (FDA) [84,140,146], and in 2019, it was also approved by the European Medicines Agency (EMA), in the treatment of seizures associated with Dravet syndrome (DS) and Lennox–Gastaut syndrome (LGS), as well as seizures associated with tuberous sclerosis [26,87,147].

The mechanisms involved in the anticonvulsant action of CBD are still unclear but may involve antagonism of G55 protein-coupled receptor (GPR55), inhibition of adenosine reuptake, and desensitization of vanilloid type 1 receptor (TRPV1) [87].

GPR55 is currently considered an orphan receptor whose activation triggers a series of events followed by intracellular Ca^2+^ release with modulation of neurotransmitter release and neuronal excitability [141]. It acts as an antagonist of GPR55, reducing the frequency, severity, and duration of spontaneous seizures, which has been demonstrated and validated in a genetic mouse model of Dravet syndrome [89].

Rett syndrome (RTT) is a rare genetic neurological disorder characterized by severe impairments affecting the ability to speak, walk, eat, and even breathe easily; it is most often diagnosed in children [143]. GPR55 was found to be increased in the Rett syndrome mouse hippocampus, suggesting that GPR55 antagonists could be potential pharmacological agents for this pathology [144]. Given that CBDV proved to have antagonistic proprieties on GPR55 [148], several studies on RTT mouse models were performed in order to study the effects of CBDV on disease symptoms. After 14 days of daily administration, CBDV proved to attenuate brain alterations, restore the compromised general status, increase sociability, and partially restore motor coordination in treated mice [144], and after 4–9 weeks of administration, CBDV delayed the appearance of neurological defects [143].

In a preclinical study using rats with autism-like behavior, created through prenatal valproic acid exposure, CBDV proved to ameliorate behavioral abnormalities, restore hippocampal endocannabinoid signaling, and decrease neuroinflammation, indicating that CBDV could be a potential therapeutic agent for autism spectrum disorders [149].

As for vanilloid type 1 receptor desensitization, CBD can modulate the intracellular Ca^2+^ concentration. The effect of TRPV1 activation and inhibition on the seizure threshold is complex, and although it is mainly a TRPV1 agonist, CBD causes rapid desensitization of the channel with a role in the antiseizure activity.

In the brain, adenosine acts as an endogenous anticonvulsant by stimulating A1 and other adenosine receptors. CBD is a potent inhibitor of the equilibrative nucleoside transporter that mediates adenosine reuptake followed by an increased extracellular concentration [147].

Another CNS pathology is Alzheimer’s disease, a condition mainly characterized by the formation of senile β-amyloid plaques and neurofibrillary tangles caused by hyperphosphorylation of tau proteins. In in vitro studies, CBD inhibited tau hyperphosphorylation and reduced Aβ production, and in in vivo studies, it reversed cognitive impairments on a double AD transgenic mouse model (APP/PS1) [26].

The acidic form of CBD was studied for its anticonvulsant effect in a mouse model of Dravet syndrome, and it was found that CBDA exhibited significant anticonvulsant properties through a mechanism that could involve the 5-HT1A, GPR55, or TRPV1 receptors [136].

It is important to note that CBD does not only act on CNS structures. Available data suggest that CBD has a potent anti-inflammatory effect, which could make it a promising candidate for various diseases, being a modulator of the immune system, causing a decrease in proinflammatory cytokines (interleukin 1-β, interleukin 6, interferon-β) in lipopolysaccharide-activated microglial cells as well as interleukin 10 and 12 in murine macrophages [150].

Recent studies have shown that CBD has promising potential in chronic, neuropathic, and inflammatory pain [82]. Cannabidiol modulates chronic neuropathic pain and depression-specific behavior by activating 5-HT1A and CB1 receptors in the prefrontal cortex, a fact which has been demonstrated in animal models. A preclinical study in Wistar rats aimed to create a model of neuropathic pain induced by sciatic nerve injury. There is a close link between chronic neuropathic pain and depression, with the prelimbic division of the medial prefrontal cortex being directly involved in both conditions. The authors found that local cannabidiol administration attenuated mechanical allodynia as well as depression-like behavior. This study showed that acute systemic administration of cannabidiol increases extracellular serotonin levels through activation of 5HT1A and CB1 in the ventromedial prefrontal cortex, involved in the regulation of emotional impairment, as cannabidiol may be proposed as a potential drug with therapeutic indications for the treatment of depressive disorders associated with chronic neuropathic pain [83].

A 2021 study concluded that CBDV and CBG (discussed previously) have neuroprotective and anti-inflammatory properties on an in vitro model of ischemic stroke obtained by exposing cells to ischemic conditions through oxygen–glucose deprivation [151].

The anti-inflammatory activity of CBDV was studied previously on an IBD mouse model of DNBS- and dextran sulfate sodium (DSS)-induced colitis. It was found that the administration of CBDV (orally or intraperitoneally) reduced the specific signs of colon inflammation–neutrophil infiltration and increased colon weight and intestinal permeability [91]. In the same study, human colonic tissues from children with active ulcerative colitis were treated in vitro with CBGV, and it was shown that this treatment produced a significant reduction in the proinflammatory cytokine levels (IL-1β) [91].

CBDA was also reported to produce anti-inflammatory and anti-hyperalgesia effects on carrageenan-induced hyperalgesia and edema in rodent models of inflammatory pain when administrated i.p. 60 min before carrageenan [135].

CBDA was also studied for its anticancer properties, and the authors reported that this compound is a potent MDA-MB-231 breast cancer cell migration inhibitor, through a mechanism that is supposed to involve the activation of RhoA (an inhibitor of cancer cell mobility), and, at the same time, the inhibition of cAMP-dependent protein kinase A [94].

In two studies, from 2013 and 2020, researchers found that CBDA can potently suppress nausea and vomiting in rats through the activation of the serotonin 1A receptor (5-HT1A) using animal models of acute lithium chloride-induced nausea [92,93].

However, CBD pharmacology is complex, affecting many different targets. It appears to bind to the same transmembrane protein site as cholesterol, interacting directly with a wide range of targets. Both cholesterol and CBD alter membrane elasticity and can specifically or non-specifically inhibit a wide range of transmembrane targets [136]. Cannabinoids such as CBD are transported to the endoplasmic reticulum by fatty acid-binding protein, the endoplasmic reticulum being the site of cholesterol homeostasis. CBD appears to alter cholesterol homeostasis by modulating the PPARγ receptor which lowers cholesterol levels by reducing HMG-reductase and increasing CYP7A1, but this mechanism is inconsistent with recent evidence that CBD can increase cholesterol levels [140].

As side effects, CBD can cause drowsiness/sedation, diarrhea, decreased appetite, rash, fatigue, sleep disturbances, increased liver transaminases, anemia, viral infections, and behavioral changes. Due to hepatic metabolism, it may cause drug–drug interactions in combination with drugs metabolized by the cytochrome P450 superfamily, such as warfarin-type anticoagulants, direct oral anticoagulants (dabigatran), antiaggregants (clopidogrel), and various antiepileptic drugs (clobazam, topiramate, zonisamide) [29,82,87,146]. In a 2019 study, Andreson et al. showed that CBD–clobazam interaction could be used to improve the anticonvulsant effect of CBD, but only when an anticonvulsant dose of CBD is used, meaning a sub-anticonvulsant dose of CBD did not potentiate the effects of clobazam, despite the presence of pharmacokinetic interaction (Anderson et al., 2019). These are not the only drug interactions of CBD, and its metabolism can be inhibited by ketoconazole (CYP3A4 inhibitor) or induced by rifampicin. Other reports suggest that CBD may increase plasma concentrations of tacrolimus and everolimus [29,82,87,146,147].

**Table 3:** The most important findings in preclinical studies on major cannabinoids and their related compounds.

Class	Compound	Experimental Model	Findings	Reference
CBG	CBG	Mouse model of intestinal inflammation induced with the intracolonic administration of DNBS	Anti-inflammatory effect associated with the downregulation of inflammatory cytokines interleukin-1β, interleukin-10, and interferon-γ and reduction in iNOS expression.	[77]
	CBG CBGA CBGV	In vitro HEK-293 cells stably overexpressing rat recombinant TRPV3 or TRPV4	CBGV and CBGA desensitize TRPV3 to the action of carvacrol at concentrations of EC50 = 0.8 and 7.4 µM. CBGV, CBGA, and CBG desensitize TRPV4 to the action of 4α-phorbol-12,13-didecanoate(4α-PDD) with EC50 values of 1.3–5.4 µM. These compounds desensitize TRPV3 and TRPV4 channels at lower doses than those at which they stimulate these channels.	[76]
	CBG CBGV	HEK-293 cells stably overexpressing human TRPV1	CBG and CBGV stimulated and desensitized human TRPV1.	[72]
	CBG CBGA	COX-1 enzyme, purified from ram seminal vesicles and COX-2 enzyme, purified from sheep placental cotyledons	Inhibition of more than 30% of COX -1 and 292 COX -2 in a concentration-dependent manner.	[78]
	CBG	Computational model of α2A, α2B, and α2C isoforms of murine and human 304 adrenoceptors	Affinity for the receptor appeared higher than that of the α2-adrenergic receptor agonist clonidine.	[152]
CBG	CBG	Mouse skin melanoma cells	Significant antitumor activity (inhibitory concentration (ICs0) = 31.31 gg/mL) in in vitro assay.	[117]
	CBG	Human oral epithelioid carcinoma 308 cell lines (KB) and NIH 3T3 fibroblasts	CBG exhibited the highest growth inhibitory activity against the cancer cell lines.	[118]
	CBG	HEK-293 encoding the rat TRPM8 and overexpressing high levels of TRPM8	Potent TRPM8 antagonist (IC50 = 0.16 ± 0.02).	[72]
CBG	CBG	Two human colon adenocarcinoma cell lines (Caco-2 and HCT 116, ATCC); Mouse azoxymethane (AOM) model of colon carcinogenesis	CBG inhibits the growth of CRC cells mainly via a pro-apoptotic mechanism and hinders the development and the growth of colon carcinogenesis in vivo.	[111]
		Mouse brain membranes	CBG activates α2-adrenoreceptors and blocks 5-HT1A receptors, antagonizing the 5-HT1A receptor agonist R-(+)-8-hydroxy-2-(di-n-propylamino) tetralin.	[86]
	CBG	TMEV (Thaler’s murine encephalomyelitis virus)-induced demyelinating disease (TMEV-IDD) in SJL/J mice	Anti-inflammatory and neuroprotective effects through the inhibition of IL-1β and IL-6 cytokines, and downregulation of PGE2 synthesis. CBG and CBG-quinone inhibited the microglia inflammatory response, protected neurons from toxic insults.	[124]
CBG	CBG	Mouse model of Huntington’s disease (HD), created using 3-Nitropropionate i.p. repeated administration	Neuroprotective effects by downregulating the proinflammatory markers COX-2, 367 iNOS, IL-6, and TNF-α, by preventing neuronal degradation, downregulating disease-associated genes SgKL and CD44, and normalizing specific protein-1 levels.	[125]
	CBG	In vitro model of neuro inflammation on NSC-34 motor neurons	Pretreatment with CBG (7.5 μM) improved viability in treated cells through the inhibition of cell apoptosis, reduction in IL-1β, TNF-α, IFN-γ, and PPAR-γ proinflammatory protein levels, reduction in oxidative stress, and upregulation of Nrf-2 levels.	[74]
	CBG	MC65 human neuron-like cell lines treated to induce intra-neuronal Alzheimer’s disease cell alterations	CBG blocked cell death, reduced oxidative damage, and prevented neurons from accumulating toxic β-amyloid protein.	[126]
	CBG	Male Lister hooded rats	Doses between 120 and 140 mg/ kg of CBG induced a dose-dependent increase in food intake, increased the number of meals taken, decreased the latency until the first meal, and improved locomotor activity.	[128]
	CBG	Standard *S. aureus* strain (ATCC 25923) and a clinical isolate (XU212) *MRSA* strain	Antibacterial properties.	[110]
		Methicillin-resistant S. aureus 404 (MRSA) strain; murine systemic infection model caused by MRSA	In vitro disruption of the cytoplasmatic membrane of MRSA. In vivo efficacy against MRSA.	[129]
	CBG	Keratinocyte proliferation assay	CBG had an inhibitory action on keratinocyte proliferation in a CB1/CB2 receptor-independent manner.	[130]
		Human keratinocytes (HaCaT cells)	CBG acted as a transcriptional repressor controlling cell proliferation and differentiation through a mechanism that involved increasing DNA methylation on the keratin-10 gene.	[131]
	CBG CBGA	Human recombinant and pig kidney aldose reductase	Both compounds showed statistically significant ALR2 inhibitory activity by being able to interact with the major active site of the enzyme.	[79]
CBG	CBG	HEK-293 cells stably overexpressing human TRPV1	Stimulates and desensitizes TRPV1 channels with an of EC50 = 21.0 ± 1.25.	[72]
		Colon cancer cells and normal colon cell lines	Cytotoxic activity on colon cancer cells, but reduced activity on normal colon cell lines.	[120]
	CBGV	HEK-293 cells encoding the rat TRPV2 and expressing high levels of TRPV2	Antagonizes TRPV2 channels with an EC50 = 1.7 μM.	[72]
CBD	CBD	Murine (mouse) model of depression	CBD reduced immobility time in mice undergoing forced swimming test, the effect being similar to that produced by antidepressants such as imipramine.	[85]
	CBD	Mouse model of autism spectrum disorders	10–20 mg/kg acute administration of CBD determined an improvement in social behavior.	[89]
	CBD	Alzheimer’s disease mouse model	20 mg/kg sub-chronic administration of CBD reversed cognitive deficits in object recognition memory and social recognition memory.	[153]
	CBD	PTSD determined by yohimbine HCl (Tocris) administration in Wistar rats	10 mg/kg acute administration came with therapeutic benefits for post-traumatic stress disorder symptoms.	[154]
	CBD	Human breast cancer cell lines MDA-MB231 and MDA-MB436	Significantly decreased Id-1 expression in metastatic breast cancer cells, leading to the downregulation of tumor aggressiveness.	[155]
CBD	CBDV	HEK-293 cells stably overexpressing human TRPV1 HEK-293 cells encoding the rat TRPV2 and expressing high levels of TRPV2 HEK-293 cells over- expressingTRPA1 HEK-293 encoding the rat TRPM8 and overexpressing high levels of TRPM8	Stimulates TRPV1 channels. Stimulates TRPV2 channels. Stimulates TRPA1 channels. Antagonizes TRPM8 channels.	[72]
		hGPR55-HEK293 cells	Antagonizes GPR55 channels.	[148]
	CBD	43-day-old rats received d,l-AMPH (4 mg/kg, i.p.) or vehicle in the conditioned place preference (CPP) paradigm (8 days), when each experimental group was re-assigned to receive CBD at two different doses (5 or 10 mg/kg, i.p) or control, for 5 days	CBD treatment prevented amphetamine relapse behavior in rats that had previously exhibited amphetamine-conditioned place preference, modulated immunoreactivity of dopaminergic targets in the prefrontal cortex and ventral striatum, areas with major involvement in drug dependence. CBD maintains dopamine transport levels.	[27]
	CBD	Mouse genetic model of Dravet syndrome (DS)	CBD reduced the frequency, severity, and duration of spontaneous seizures through the antagonization of GPR55 receptors.	[89]
		Mecp2 mutant mice, a model of Rett syndrome (RTT)	CBDV rescues recognition memory deficits in Mecp2 mutant mice and delays the appearance of neurological defects.	[149]
		Mouse model for Rett syndrome, caused by mutations in the MECP2 gene	CBDV proved to attenuate brain alterations, restore the compromised general status, increase sociability, and partially restore motor coordination in treated mice. Molecularly, CBDV has antagonistic properties on GPR55.	[144]
	CBD	Double AD transgenic mouse model (APP/PS1)	CBD inhibited tau hyperphosphorylation and reduced Aβ production.	[26]
CBD	CBD	Wistar rat model of neuropathic pain (Bennet and Xie’s NP model (1988))	CBD modulates chronic neuropathic pain and depression-specific behavior by activating 5-HT1A and CB1 receptors in the prefrontal cortex.	[83]
	CBDV	Autism-like behavior models through prenatal valproic acid exposure in rats	CBDV ameliorated behavioral abnormalities, restored hippocampal endocannabinoid signaling, and decreased neuroinflammation.	[149]
	CBDV	In vitro model of ischemic stroke obtained by exposing cells to ischemic conditions through oxygen–glucose deprivation	CBDV has neuroprotective and anti-inflammatory properties.	[151]
	CBDV	IBD mouse model of DNBS- and DSS-induced colitis	CBDV (orally or intraperitoneally) reduced the specific signs of colon inflammation–neutrophil infiltration, and increased colon weight and intestinal permeability.	[91]
		Human colonic tissues from children with active ulcerative colitis	In vitro treatment with CBGV produced a significant reduction in the proinflammatory cytokine levels (IL-1β).	
	CBDA	Mouse model of Dravet syndrome (Scn1aRX/+ mice)	CBDA exhibited significant anticonvulsant properties through a mechanism that could involve the 5-HT1A, GPR55, or TRPV1 receptors.	[136]
		Rodent models of carrageenan-induced inflammatory pain	I.p. administration of CBDA at 60 min before carrageenan produced anti-inflammatory and anti-hyperalgesia effects.	[135]
		MDA-MB-231 breast cancer cell model	CBDA inhibited cell migration through a mechanism that is supposed to involve the activation of RhoA and through the inhibition of cAMP-dependent protein kinase A.	[94]
		Rat models of acute lithium chloride-induced nausea	CBDA suppresses nausea and vomiting in rats through the activation of the serotonin 1A receptor (5-HT1A).	[92,93]
THC	Δ9-THC	Murine model of concanavalin A (ConA)-induced hepatitis	Intraperitoneal administration of THC inhibited hepatitis by significant decrease in liver enzymes and reduced liver tissue injury. THC treatment significantly suppressed inflammatory cytokines in ConA-induced hepatitis.	[156]
	Δ9-THC	Splenocytes of C57BL/6 mice	In vitro THC treatment significantly reduced proliferative response to mitogens, including anti-CD3 monoclonal antibodies (mAbs), concanavalin A (Con A), and lipopolysaccharide (LPS).	[157]
	Δ9-THC	Sprague Dawley male rats	Δ9-THC therapy inhibited acetylcholinesterase, reduced amyloid-β levels and hippocampal neurogenesis, and induced brain-derived neurotrophic factor release through mixed CB1 and CB2 modulation.	[9,117]
	Δ9-THC	Genes encoding human, mouse, and rat TRPV2	Δ9-THC is a potent TRPV2 agonist.	[101]
	Δ8-THC	Water-deprived albino rats	Groups treated with 5.0 and 10.0 mg/kg of Δ8-THC reduced intake of food at 1 day post-injection.	[158]
	THCV	Rat recombinant TRPV3- and TRPV4-expressing HEK-293 cells	Stimulates TRPV3 with high efficacy (50–70% of the effect of ionomycin) and potency (EC50 = 3.7 μM) and TRPV4 with moderate-high efficacy (30–60% of the effect of ionomycin) and potency (EC50 = 0.9–6.4 μM) [76].	[76]
	Δ9-THCA	HEK-293T, Neuro-2a (N2a), STHdh Q7/Q7, and STHdh Q111/Q111 cells, which express either a wild-type or a mutated form of the huntingtin protein	Δ9-THCA activated PPARγ and increased mitochondrial mass in neuroblastoma N2a cells and prevented cytotoxicity induced by serum deprivation in STHdh Q111/Q111 cells and by mutHtt-q94 in N2a cells. Δ9-THCA showed potent neuroprotective activity, worth consideration for the treatment of Huntington’s disease and possibly other neurodegenerative and neuroinflammatory diseases.	[104]
	Δ9-THCA-A	Mouse model of HFD significantly induced obesity	Administration of Δ9-THCA-A reduced fat mass and body weight gain, markedly ameliorating glucose intolerance and insulin resistance, and largely preventing liver steatosis, adipogenesis, and macrophage infiltration in fat tissues.	[159]

CBG, cannabigerol; CB1, cannabinoid receptor 1; CB2, cannabinoid receptor 1; TRPM8, Transient Receptor Potential Melastatin-8; TRPV1, vanilloid receptor 1; α2-Adrenoceptor, alpha-2-Adrenoceptor; IL-1β, interleukin-1β; TNF-α, tumor necrosis factor alpha; IFN-γ, interferon gamma; PPAR-γ, peroxisome proliferator-activated receptor gamma; Nrf-2 levels, nuclear factor E2-related factor 2; TRPA1, transient receptor potential ankyrin 1; TRPV3, transient receptor potential vanilloid-3; TRPV4, transient receptor potential vanilloid-type 4; iNOS expression, inducible nitric oxide synthase expression; CBGV, cannabigerovarin; CBGA, cannabigerolic acid; CBD, cannabidiol; CBDV, cannabidivarin; CBDA, cannabidiolic acid; COX-1, COX-2, Cyclooxygenase-1, Cyclooxygenase-2; SOD, superoxide dismutase; PLA2, Phospholipase A2; MAGL, monoacylglycerol lipase; PPARα/γ, peroxisome proliferator-activated receptors α/γ; GPR55, G protein-coupled receptor 55; CBDV, cannabidivarin; TRPV2, transient receptor potential vanilloid 2; GPR6, G Protein-Coupled Receptor 6; DAGLα, diacylglycerol lipase-alpha; AEA, N-arachidonoylethanolamine (anandamide); 5HT1A, 5-hydroxytryptamine receptor 1A; cAMP protein kinase A, cyclic adenosine monophosphate protein kinase A; THC, tetrahydrocannabinol; Δ9-THC, Δ9-trans-tetrahydrocannabinol; MDSCs, myeloid-derived suppressor cells; AchE, acetylcholinesterase; Δ8-THC, Δ8-trans-tetrahydrocannabinol; THCA-A, tetrahydrocannabinolic acid; IBD, inflammatory bowel disease; ConA, concanavalin A; mAbs, monoclonal antibodies; LPS, lipopolysaccharide; i.p., intraperitoneal; DNBS, dinitrobenzene sulphonic acid; DSS, dextran sulfate sodium; AD, Alzheimer’s disease.

**Table 4:** The most important findings in clinical studies on major cannabinoids and their related compounds.

Class	Compounds	Clinical Study	Results	Reference
CBD THC	CBD THC THC + CBD	A 4-way, double-blind, placebo-controlled crossover design study in cannabis users. 48 volunteers, CBD (16 mg), THC (8 mg), THC + CBD (8 mg + 16 mg), and placebo, by inhalation.	CBD improved emotional facial affect recognition at 60% emotional intensity. THC was detrimental to the recognition of ambiguous faces of 40% intensity. THC alone and combined THC+CBD equally increased feelings of being “stoned”.	[145]
CBD	CBD	Double-blind, placebo-controlled trial. 120 children and young adults with the Dravet syndrome and drug-resistant seizures, CBD oral solution, 20 mg/kg of body weight/day or placebo, in addition to standard antiepileptic treatment.	The median frequency of convulsive seizures per month decreased from 12.4 to 5.9 with cannabidiol, as compared with a decrease from 14.9 to 14.1 with placebo. The percentage of patients who had at least a 50% reduction in convulsive seizure frequency was 43% with cannabidiol and 27% with placebo.	[160]
	CBD	Double-blind, randomized clinical trial in 199 children with Dravet syndrome on cannabidiol (10 or 20 mg/kg/d) or matched placebo for 14 weeks.	Convulsive seizure frequency compared with baseline was reduced by 48.7% in the 10 mg/kg/d cannabidiol group and 45.7% in the 20 mg/kg/d cannabidiol group vs. 26.9% in the placebo group.	[161]
	CBD	Double-blind, placebo-controlled trial conducted at 30 clinical centers; we randomly assigned patients with Lennox–Gastaut syndrome. 225 patients were enrolled; 76 patients were assigned to the 20 mg cannabidiol group, 73 to the 10 mg cannabidiol group, and 76 to the placebo group.	The median percent reduction from baseline in drop seizure frequency during the treatment period was 41.9% in the 20 mg cannabidiol group, 37.2% in the 10 mg cannabidiol group, and 17.2% in the placebo group.	[162]
	CBD	Double-blind, placebo-controlled, randomized crossover trial in 39 healthy young subjects. A single dose of cannabidiol e-liquid (0.25 mL, 5% cannabidiol, 12.5 mg cannabidiol) and once placebo for vaping after learning 15 unrelated nouns.	Cannabidiol enhanced verbal episodic memory performance (placebo: 7.03 [2.34]; cannabidiol 7.71 [2.48]).	[163]
	CBDV	Case–control, placebo-controlled, randomized, double-blind, repeated-measures, crossover study on 34 subjects with autism spectrum disorder.	CBDV shifts subcortical levels of the brain’s primary excitatory metabolite glutamate both in the neurotypical and autistic brain; however, there may be significant response variability in ASD.	[142]

#### 2.2.3. Tetrahydrocannabinol (THC)-Type Compounds

Tetrahydrocannabinol or THC, as it is abbreviated, is the main psychoactive element of *Cannabis* and one of the 125 recognized cannabinoids in the plant *Cannabis sativa*. The term THC generally refers to the delta-9-THC isomer with the chemical name ∆9-tetrahydrocannabinol (∆9-THC) isolated from a hexane extract of hashish in 1964 by Goani and Mecholum. Other than ∆9-THC, tetrahydrocannabivarin (THCV, isolated in 1971, from a *Cannabis* tincture of Pakistani origin), ∆9-tetrahydrocannabinolic acid A (∆9-THCA-A, isolated in 1967), and Δ8-trans-tetrahydrocannabinol (Δ-8-THC, isolated in 1966, from the flowers and leaves of a plant grown in Maryland) are major cannabinoids also identified in *Cannabis sativa* [117].

In healthy people, Δ9-THC can cause acute psychotic reactions, together with a temporary decline in both cognitive function [9] and psychomotor control [164]. In patients with schizophrenia, Δ9-THC may intensify memory impairments, psychotic symptoms, and anxiety [150]; additionally, several studies have indicated a relationship between systematic *Cannabis* use and an increased risk of developing this condition [149]. Currently, there is growing preclinical evidence that Δ9-THC can suppress inflammation by activating CB2 or CB1/CB2 through multiple pathways, including: shifting the balance of human T helper 1 (Th1)/T helper 2 (Th2) cells [95], T reg differentiation [140], myeloid-derived suppressor cell induction (MDSC) [151], generation of apoptosis in dendritic and activated T cells [156,157], or induction of immunosuppressive MDSCs and T regs [165]. In Alzheimer’s disease, evidence suggests that Δ9-THC therapy may inhibit acetylcholinesterase [166], reduce amyloid-β levels [99] and hippocampal neurogenesis [100], and induce brain-derived neurotrophic factor release through mixed CB1 and CB2 modulation [96,167]. dditionally, in clinical and preclinical studies, ∆9-THC has a series of essential therapeutic benefits, such as analgesia, appetite stimulation, and antiglaucoma and antiemetic effects facilitated by both CB1 and/or CB2 activation [97,168]. However, the therapeutic use of ∆9-THC is restricted by its psychoactivity and potential for inducing tolerance and dependence. The neural basis for these divergent effects of Δ9-THC on cognitive function and psychiatric symptoms is unclear. Recent data from in vivo and in vitro studies suggest that Δ9-THC may have opposite effects than those mentioned above on cerebral CB1 receptors mediated by a partial agonism [8]. Moreover, ∆9-THC is also characterized as a partial agonist of CB2 [169,170]. As a characteristic partial agonist, ∆9-THC has a combined agonist–antagonist effect which is likely dependent on receptor expression, cells, and the simultaneous presence of endocannabinoids or other agonists [171]. The affinity of Δ-9-THC for CB1 and CB2 receptors (KiCB1 = 5.05 nM, KiCB2 = 3.13 nM [170]; KiCB1 = 35.3 nM, KiCB2 = 3.9 nM [172]; KiCB1 = 39.5 nM, KiCB2 = 40 nM [173]; KiCB1 = 21 nM, KiCB2 = 36.4 nM [174]; KiCB1 = 36 nM, KiCB2 = 31 nM [175]) exceeds or matches that of the phytocannabinoids THCV (KiCB1 = 75.4 nM, KiCB2 = 62.8; KiCB1 = 22 nM, KiCB2 = 47 nM [176]) and Δ-8-THC (KiCB1 = 44 nM, KiCB2 = 44 nM [175]; KiCB1 = 47.6 nM, KiCB2 = 39.3 nM [177]).

Δ8-THC is chemically more stable than Δ9-THC and also exhibits psychoactive effects. As with Δ9-THC, it may behave as a CB1 receptor antagonist and partial CB2 agonist [175], exhibiting similar properties in in vitro and in vivo studies: behaving as a conjugate for some fatty acids, reducing the signs of inflammation and inflammatory pain in mice, or showing inhibitory dose-dependent effects on water intake with implications for behavioral studies [178].

The lack of cannabimimetic effects has made THCA-A a more attractive compound, leading to an increase in interest in its use in the clinic [179]. Rock et al. [180] suggested that the lack of psychoactivity may be due to limited access to CB1R. Affinity and efficacy studies of THCA-A at cannabinoid receptors revealed disparate results—equal to [181] or 25-fold weaker than Δ9-THC [69], or non-affinity [182,183]. Rosenthaler et al. [69] determined a Ki of 23.4 nM for THCA-A on CB1R, almost equivalent to that for Δ9-THC (Ki = 35.6 nM), according to data of a Δ9-THC meta-analysis, which described a mean Ki of 25.1 ± 0.39 nM at CB1 (*n* = 16 studies) [68]. Verhoeckx et al. [182] reported a Ki of 890 nM for THCA-A at CB1R and a Ki of 3.5 nM for THC at CB1R, which is 7-fold higher than the meta-analytic mean. In line with data suggesting low affinity, THCA-A displayed low efficacy at CB1. THCA-A (10 μM) may cause a small but substantial forskolin cAMP inhibition, consistent with agonist activity. Regarding CB2, in cAMP assays, THCA-A did not produce any significant effect [179].

Pharmacological data suggest that tetrahydrocannabinol-type compounds target more than canonical cannabinoid receptors [171,184].

Δ9-THC did not display a response at TRPV1, while several studies describe its agonistic effects at the TRPV2, TRPV3, and TRPV4 channels. Using a cell-based calcium mobilization assay and patch clamp electrophysiological evaluation, Neeper et al. [185] were able to identify Δ9-THC as a novel TRPV2 agonist. However, Δ9-THC exhibits poor selectivity and may also activate TRPA1 [101].

There are not many data on Δ8-THC activity at other targets, such as GPR18, GPR55, PPARγ nuclear receptors, or TRP channels. However, it is believed that this compound has a pharmacological profile similar to ∆9-THC [186].

THCV has been shown to stimulate TRPV3 with high efficacy (50–70% of the effect of ionomycin) and potency (EC_50_ = 3.7 μM) and TRPV4 with moderate-high efficacy (30–60% of the effect of ionomycin) and potency (EC_50_ = 0.9–6.4 μM) [158], by assessing [Ca2^+^] elevation in rat recombinant TRPV3- and TRPV4-expressing HEK-293 cells. THCV (potency shown as EC_50_ 1.5 ± 0.2 µM) also stimulated human TRPV1 [76].

In vitro functional tests and docking analysis showed that THCA-A binds to and stimulates PPARγ by acting at both the alternative and the canonical sites of the ligand-binding domain, being at least 20-fold more potent than Δ9-THC [187]. Transcriptomic signatures, immunohistochemistry, and plasma biomarker analyses from a mouse model of high-fat diet- induced obesity treated with THCA-A has been shown to reduce fat mass and gain in body weight, significantly improve glucose intolerance and insulin resistance, and largely prevent adipogenesis, macrophage infiltration, and hepatic steatosis. Additionally, THCA-A therapy caused browning of inguinal white adipose tissue and displayed potent anti-inflammatory actions [104]. Investigating the in vivo neuroprotective activity of THCA-A in mice intoxicated with the mitochondrial toxin 3-NPA, Nadal et al. [104] showed that THCA-A therapy attenuated astrogliosis, microgliosis, and upregulation of proinflammatory markers caused by 3-NPA through a PPARγ-dependent pathway.

The therapeutic potential of these compounds still remains largely unanswered which underlines the need for subsequent preclinical and clinical research to highlight whether these compounds can really be “a neglected pharmacological treasure trove”.

## 3. Structure Modulation to Obtain New Pharmacological Effects

THC is the most explored compound of its class, being the first, in the attempt to obtain new therapeutic molecules targeting the endocannabinoid system. The group of new compounds was named “synthetic cannabinoids” and has emerged because of the need to accurately explore the endocannabinoid system and to obtain new potent therapeutic resources for pain, neurodegenerative diseases, emesis, obesity, and cancer, with minor or even absent side effects. In 1979, in an attempt to develop new analgesics, Pfizer developed several synthetic THC analogs such as CP47497 (2-[(1S, 3R) -3-hydroxycyclohexyl]-5-(2-methyloctan-2-yl) phenol) and the more potent compound CP55940 (2 [(1R, 2R, 5R)-5-hydroxy-2-(3-hydroxypropyl)-cyclohexyl]-5-(2-methyloctan-2-yl) phenol). These compounds have shown analgesic effects, but also marked neurological adverse effects due to the increased affinity for the CB1 receptor revealed subsequently in experimental animal studies [159].

In 1988, at the Hebrew University of Jerusalem, one of the first synthetic cannabinoids, the THC analog called HU-210, was synthesized. According to preliminary results, the drug showed strong analgesic properties, but also sleep-inducing effects, 100 times greater than those of THC [188].

In an attempt to develop high-affinity compounds that specifically target only one type of cannabinoid receptor, the first CB1 antagonist named SR141716A-Rimonabant (N-piperidino-5-(4-chlorophenyl)-1-(2,4-dichlorophenyl)-4-methyl-1H-pyrazole-3-carboxamide), with a selectivity of over 1000 x for CB1 over CB2, was synthesized in 1994. Of note, this drug had the ability to inhibit the psychoactive effect. Similarly, the first inverse CB2 agonist named SR144528 (N-[(1S,2S,4R)-1,3,3-trimethylbicyclo[2.2.1] heptan-2yl]-5-(4-chloro-3-trimethylphenyl)-4-methyl-1-(4-methylphenyl)-1 H-pyrazole-3-carboxamide) was synthesized in 1998 [189]. Another compound with analgesic potential is the CB2 receptor-selective agonist named HU-308 [173].

Experimental animal tests have shown that blocking CB1 decreases appetite and may improve the evolution of neurodegenerative and psychiatric diseases (Alzheimer’s disease, schizophrenia, multiple sclerosis). On the other hand, CB1 stimulation has antiemetic, analgesic, cardioprotective, and antineoplastic effects, while CB2 stimulation has anti-inflammatory and immunomodulatory properties [190].

The first attempts to structurally manipulate the structure–affinity relationship in the cannabinoid system were performed on classical cannabinoid analogs (THC), with a focus on the modification of the C3 side chain. Thus, it was observed that the increased length of the C3 side chain correlates directly with both the increase in affinity for CB1 and CB2 and vice versa [191]. After rigorous research on the length of the alkyl group required for binding affinity, it was established that the optimal chain length is eight carbon atoms and the minimum chain length is three carbon atoms, in order to obtain binding affinity for CB receptors [192]. Since then, numerous experiments have been carried out with the aim of modifying the chemical structure in order to obtain compounds with great affinity even several thousand times higher than the classic natural extracts [193].

The history of important scientific discoveries has shown that results can be interpreted and used both to make a beneficial contribution to society or to cause harm (e.g., research on the atom and atomic energy, or opioids). Therefore, research on the manipulation of the cannabinoid system is no exception in this regard.

As early as the 2000s, synthetic compounds (popularly called spice) began to be produced to be sold as psychotropic recreational drugs on the black market. Laboratory tests performed on various samples of products sold in the drug market revealed the presence of synthetic cannabinoids in various mixtures and concentrations, such as JWH-018 (an agonist of both cannabinoid receptors), and CP47497-C8 (potent agonist of CB1) [192].

The uncontrolled and unregulated use of these substances has led to significant psychiatric side effects, accidents, and highly addictive behavior, which is the reason why most of the European countries have banned them since 2009. The ban and cataloging of these compounds as high-risk drugs have not stopped the production of many other synthetic analogs and their illegal marketing, according to the European Drug Report from 2021 [194].

For these reasons, research on cannabinoids derived from THC has been heavily hampered by the stigma of high-risk banned drugs. However, there is clear evidence that cannabinoid-derived compounds can bind different types of receptors and exert their effect through mechanisms that do not necessarily involve the endocannabinoid system, which could be useful in treating several challenging conditions.

**Figure 2:**
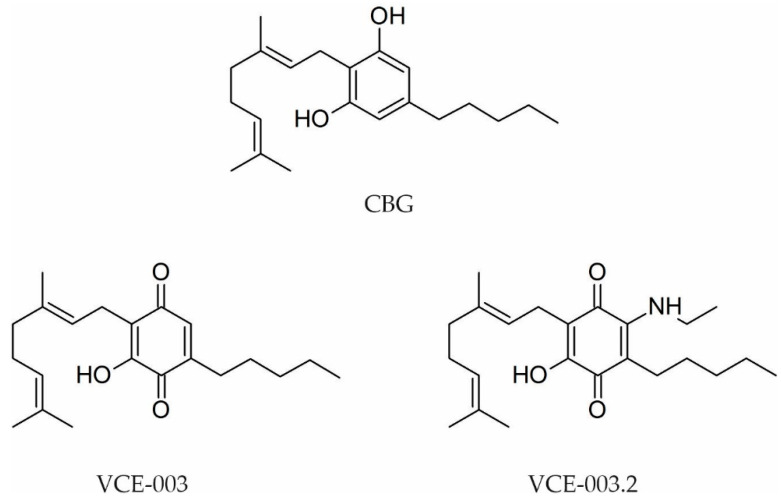
Chemical structures of CBG derivatives VCE-003 and VCE-003.2.

Knowing that PPARγ activity can be increased by natural cannabinoids such as Δ9-THC and CBD [195,196], Granja et al. conducted a study in 2012 to test the activity of CBG and its quinone derivative VCE-003 (Figure 2) on a multiple sclerosis model produced with Theiler’s murine encephalomyelitis virus (TMEV). The authors demonstrated that VCE-003 is more potent than CBG on neuronal cell protection from excitotoxicity, and that VCE-003 increased PPARγ activity, inhibited the release of proinflammatory mediators, and decreased microglial reactivity. In the same study, the authors reported that VCE-003 has no affinity for the CB1 receptor, but it is ten times more potent in binding the CB2 receptor than CBG [115]. In 2014, the same group of researchers reported that VCE-003 had immunosuppressive and anti-inflammatory activities through the activation of PPARs and CB2 receptors in an in vivo model of experimental autoimmune encephalomyelitis (EAE). These two studies demonstrated that this quinone derivative of CBG is a promising therapeutic agent for the treatment of diseases with inflammatory and autoimmune components, such as multiple sclerosis or autoimmune encephalomyelitis [197].

**Figure 3:**
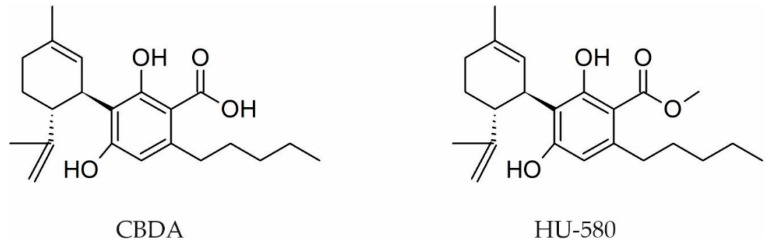
Chemical structure of CBDA derivative HU-580.

Going further, Alonzo et al. synthetized the analog compound VCE-003.2 (Figure 3) starting from VCE-003 and tested for neuroprotective actions on an animal model of Huntington’s disease induced by quinolinic acid (QA) and 3-NPA administration. VCE-003.2 administration improved motor deficits, inhibited the upregulation of proinflammatory markers, prevented medium spiny neuronal loss, and improved antioxidant defense in this in vivo model of HD [198]. In another study from 2018, the activity of VCE-003.2 was tested on an experimental model of Parkinson’s disease. The authors demonstrated that this compound has no activity at CB receptors but has neuroprotective activity against neuronal damage caused by inflammation, through the activation of PPARγ receptors, and by reducing elevated levels of proinflammatory mediators such as TNF-α, IL-1β, COX-2, and iNOS [199].

The compound continued to be tested with good results on an experimental model of amyotrophic lateral sclerosis, and it was found that i.p. administration of 10 mg/kg successfully improved neuropathological deterioration and normalized IL-1β levels, an effect that could involve PPAR-γ activation [200].

In 2019, Aguareles et al. and Burgas et al. tested VCE-003.2 oral administration on animal models of HD and experimental models of Parkinson’s disease. The conclusion was that oral administration of VCE-003.2 protected striatal medium spiny neurons from mutant damage produced by HD, attenuated neuroinflammation, and improved motor performance [199]. In the PD model, oral administration of 20 mg/kg of VCE-003.2 protected against neuronal damage caused by inflammation [201].

Another attempt to modulate the structure of CBG was made by Annalisa Lopatriello in 2018, whose team of researchers performed an iodine-mediated cyclization of cannabigerol in an attempt to expand the pharmacological and chemical space of this major phytocannabinoid. The resulting compounds were tested for their activity on transient receptor potential melastatin (TRPM) receptors starting from the premise that they could have improved action on these receptors because CBG is a potent agonist of TRPMA1 and a potent antagonist of TRPM8 [72,75]. Experiments have shown that all cyclic compounds have an increased activity on TRPMA1 and a slightly improved affinity for TRPM8, which makes them better candidates for future tests as pharmacological agents in diseases with neuropathic and/or inflammatory pain [75].

Regarding cannabidiol, four authors reported results for a new derivative compound, cannabidiolic acid methyl ester (HU-580), that was tested in animal models of depression, neuropathic pain, anxiety, and nausea [202,203,204,205]. The authors found that HU-580 reduces stress-induced depression-like behavior in rats and provides antinociception in a model of peripheral neuropathic pain for male rats, with no efficacy in females [203,204]. Studied for its 5HT1A receptor-mediated activity, it was found that, in vitro, HU-580 is more potent than CBDA at enhancing 5-HT1A receptor activation and, in vivo, reduces nausea and anxiety in acute and anticipatory nausea models developed on rats [205]. In another experiment, CBD, CBDA, and HU-580 were administrated daily for one and four weeks. It seemed that the compounds maintained their effectiveness in reducing LiCl-induced vomiting and nausea in rats and shrews. Thus, these three compounds can be taken into consideration as pharmacological agents for the treatment of nausea in chronic conditions, without the risk of developing tolerance [205].

## 4. Conclusions

The multifaceted aspects of the adaptive pro-homeostatic physiological or maladaptive pathological roles of the endocannabinoid system and major phytocannabinoids—which were originally neglected—are becoming important again. Exploring the landscape of major phytocannabinoids and their derivatives beyond the cannabinoid receptors may lead to a better understanding of human physiology, which will help us to develop new and more selective compounds to better realize the therapeutic potential of cannabis in disorders with or without a multifactorial nature.

## Figures and Tables

**Table 1:** Classification of phytocannabinoids. Adapted from [9], MDPI, 2021.

Class of Compounds	The Number of Compounds in Each Class	The First Representative Compound of the Class	Chemical Structure of the Representative Compound
Δ9-trans-tetrahydrocannabinol	25	Δ9-THC—isolated in 1964 by Goani and Mecholum using chromatography techniques [10]	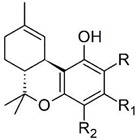
Δ8-trans-tetrahydrocannabinol	5	Δ8-THC—isolated in Maryland in 1966 [11]	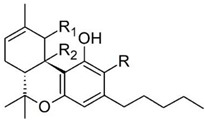
cannabidiol	10	CBD-C5—isolated in 1940 from native Minnesota hemp [12]	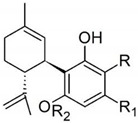
cannabigerol	16	CBG—isolated in 1964 using florisil chromatography [13]	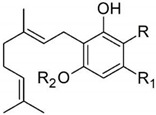
cannabichromene	9	CBC—isolated in 1966 by Gaoni Y. [14]	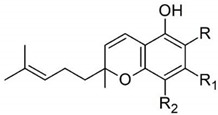
cannabinol	11	CBN synthesized by Adams et al. in the US and by Todd’s group in the UK in 1940 [15,16]	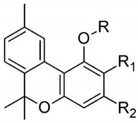
cannabinodiol	2	CBND-C3—isolated in 1973 [17] CBND-C5—isolated in 1977 [18]	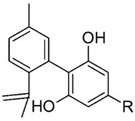
cannabicyclol	3	CBL—compound was isolated by Korte and Sieper in 1964, and the structure was elucidated by Crombie et al. in 1968 [19,20]	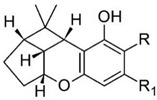
cannabielsoin	5	CBE-C5—isolated in 1973 from Lebanese hashish [21]	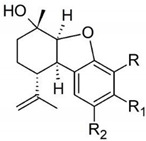
cannabitriol	9	CBT-C5—isolated in 1966 from Japanese hemp, but the complete chemical structure was established 10 years later [21,22]	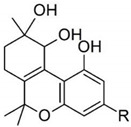
other unclassified types of cannabinoids	30	The first ones isolated in 1975 (examples: dehydrocannabifuran DCBF-C5, cannabifuran CBF-C5) [23]	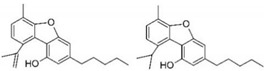

**Table 2:** Major cannabinoid targets.

Class	Compounds	Targets	Effects	Potential Use as/in	References
CBG	CBG	CB1	Poor agonist		[70]
		CB2	Partial agonist		[70]
		TRPM8	Antagonist	Prostatic cancer	[72]
		TRPV1	Stimulation	Pain and inflammation, breast, skin, colon cancer	[72]
		α2-Adrenoceptor	Agonist	Anti-inflammatory	[25,73]
		IL-1β	Reduction	Neuroinflammation	[74]
		TNF-α			
		IFN-γ			
		PPAR-γ			
		Nrf-2 levels	Upregulation		
	CBG, cyclic CBG	TRPA1	Activation	Analgesic, anti-inflammatory	[75]
	CBG, CBGV, CBGA	TRPV3 TRPV4	Activation and desensitization	Anti-inflammatory agent in IBD	[76]
		iNOS expression	Reduction	Anti-inflammatory	[77]
		SOD	Increased activity		
		Cytokines	Downregulation		
	CBG, CBGA	COX-1, COX-2	Inhibition	Anti-inflammatory	[78]
		PLA2	Inhibition		
		MAGL	Inhibition		
	CBG CBGA	ALR	Inhibition	Diabetic complications	[79]
		PPARα/γ	Full or partial agonist		[80]
	CBGV	TRPV2	Antagonist	Cancer	[72,81]
CBD	CBD	CB1	Activation	Chronic neuropathic pain	[82,83]
		TRPV1	Agonist	Depression	[84,85]
		5HT1A	Agonist		
		PPARγ	Agonist		
	CBDV	CB1/CB2	Indirect inhibition		[86]
		TRPA1	Stimulation		[72]
		TRPV1	Desensitization		[87]
		TRPV2	Stimulation		[88]
		GPR55	Antagonist	Dravet syndrome, anticonvulsant	[87,89]
		GPR6	Inverse agonist		[90]
		DAGLα	Inhibition		[86]
		AEA	Inhibition of cellular uptake		[86]
		IL-1β	Reduction	IBD	[91]
	CBDA	5HT1A	Activation	Nausea	[92,93]
		cAMP protein kinase A	Inhibition	Breast cancer	[94]
THC	Δ9-THC	CB1/CB2	Activation	Anti-inflammatory	[95]
			Mixed modulation	Alzheimer	[96,97]
		MDSCs	Induction	Anti-inflammatory	[98]
		AchE	Inhibition	Alzheimer	[99]
		Amyloid-β	Reduction	Alzheimer	[100]
		TRPV2	Agonist		[101,102]
		TRPV3	Agonist		[76,101]
		TRPV3	Agonist		[76,101]
	Δ8-THC	CB1	Antagonist	Anti-inflammatory	[103]
		CB2	Partial agonist	Mood disorders	[103]
	THCA-A	PPARγ	Stimulation	Obesity	[104]

CBG, cannabigerol; CB1, cannabinoid receptor 1; CB2, cannabinoid receptor 1; TRPM8, Transient Receptor Potential Melastatin-8; TRPV1, vanilloid receptor 1; α2-Adrenoceptor, alpha-2-Adrenoceptor; IL-1β, interleukin-1β; TNF-α, tumor necrosis factor alpha; IFN-γ, interferon gamma; PPAR-γ, peroxisome proliferator-activated receptor gamma; Nrf-2 levels, nuclear factor E2-related factor 2; TRPA1, transient receptor potential ankyrin 1; TRPV3, transient receptor potential vanilloid-3; TRPV4, transient receptor potential vanilloid-type 4; iNOS expression, inducible nitric oxide synthase expression; CBGV, cannabigerovarin; CBGA, cannabigerolic acid; COX-1, COX-2, Cyclooxygenase-1, Cyclooxygenase-2; SOD, superoxide dismutase; PLA2, Phospholipase A2; MAGL, monoacylglycerol lipase; PPARα/γ, peroxisome proliferator-activated receptors α/γ; GPR55, G protein-coupled receptor 55; CBDV, cannabidivarin; TRPV2, transient receptor potential vanilloid 2; GPR6, G Protein-Coupled Receptor 6; DAGLα, diacylglycerol lipase-alpha; AEA, N-arachidonoylethanolamine (anandamide); 5HT1A, 5-hydroxytryptamine receptor 1A; cAMP protein kinase A, cyclic adenosine monophosphate protein kinase A; THC, tetrahydrocannabinol; Δ9-THC, Δ9-trans-tetrahydrocannabinol; MDSCs, myeloid-derived suppressor cells; AchE, acetylcholinesterase; Δ8-THC, Δ8-trans-tetrahydrocannabinol; THCA-A, tetrahydrocannabinolic acid; IBD, inflammatory bowel disease.

## Data Availability

No new data were created or analyzed in this study. Data sharing is not applicable to this article.

## Abbreviations

THC, tetrahydrocannabinol; Δ9-THC, Δ-9-tetrahydrocannabinol; Δ8-THC, Δ-8-tetrahydrocannabinol; THCA-A, Δ-9-tetrahydrocannabinolic acid; CBD, cannabidiol; CBG, cannabigerol; CBN, cannabinol; CBDV, cannabidivarin; CBC, cannabichromene; CBND, cannabinodiol; CBE, cannabielsoin; CBL, cannabicyclol; CBT, cannabitriol; DCBF, dehydrocannabifuran; CBF, cannabifuran; DNBS, dinitrobenzene sulphonic acid; Th1 cells, human T helper 1 cells; Th2 cells, human T helper 2 cells; CB1, cannabinoid receptor 1; CB2, cannabinoid receptor 1; TRPM8, Transient Receptor Potential Melastatin-8; TRPV1, vanilloid receptor 1; α2-Adrenoceptor, alpha-2-Adrenoceptor; IL-1β, interleukin-1β; TNF-α, tumor necrosis factor alpha; IFN-γ, interferon gamma; PPAR-γ, peroxisome proliferator-activated receptor gamma; Nrf-2 levels, nuclear factor E2-related factor 2; TRPA1, transient receptor potential ankyrin 1; TRPV3, transient receptor potential vanilloid-3; TRPV4, transient receptor potential vanilloid-type 4; iNOS expression, inducible nitric oxide synthase expression; COX-1, COX-2, Cyclooxygenase-1, Cyclooxygenase-2; SOD, superoxide dismutase; PLA2, Phospholipase A2; MAGL, monoacylglycerol lipase; PPARα/γ, peroxisome proliferator-activated receptors α/γ; GPR55, G protein-coupled receptor 55; TRPV2, transient receptor potential vanilloid 2; GPR6, G Protein-Coupled Receptor 6; DAGLα, diacylglycerol lipase-alpha; AEA, N-arachidonoylethanolamine (anandamide); 5HT1A, 5-hydroxytryptamine receptor 1A; cAMP protein kinase A, cyclic adenosine monophosphate protein kinase A; AchE, acetylcholinesterase; IBD, inflammatory bowel disease; ConA, concanavalin A; mAbs, monoclonal antibodies; LPS, lipopolysaccharide; i.p., intraperitoneal; DNBS, dinitrobenzene sulphonic acid; DSS, dextran sulfate sodium; AD, Alzheimer’s disease.
